# An indigenous dataset for the detection and classification of apple leaf diseases

**DOI:** 10.1016/j.dib.2024.110165

**Published:** 2024-02-06

**Authors:** Arshad Ahmad Yatoo, Amit Sharma

**Affiliations:** School of Computer Applications, Lovely Professional University, India

**Keywords:** Apple leaf dataset, Automated disease detection, Phytopathology

## Abstract

Like other crops, different types of diseases affect apple trees. These diseases cause ugly cosmetic changes on the fruit and hence reduce its shelf life and value. To eliminate their impact, they need to be detected well in advance before any control measures are applied. The manual method of disease detection and subsequent classification has flaws as it involves manual scouting and analysis of the affected leaves through the naked eye. Besides, the manual method may result in wrong judgment as the knowledge of an expert limits the accuracy. Deep Learning Models have been successfully implemented for automated disease detection and classification. However, these models need massive datasets for training, testing and validation. This study proposes one such dataset that has been built indigenously by collecting images from the apple cultivation fields of Kashmir valley and subjecting it to cleaning and subsequent annotation by experts. Augmentation techniques have been used to enhance the size and quality of the dataset to prevent over-fitting of deep learning models.

Specification Table

**Table:** 

Subject	Computer Science
Specific Subject Area	Computer Science Applications, Computer Vision and Pattern Recognition
Data Format	Raw, Analyzed
Type of Data	Image
Data Collection	The dataset presented in this article contains images of healthy leaves and leaves with the symptoms of Apple-Mosaic and Alternaria. Images were captured from apple cultivation fields in Kashmir Valley. The diversity has been added by capturing the images using different devices and under different lighting conditions, e.g., sunny days, cloudy days and rainy days. To incorporate all possible conditions encountered in the actual field, the following caution has been exercised while recording the data; • Images of a particular disease or class have been collected at different stages of symptom development. • As the shape, size, and texture of a leaf varies from one variety of apple to another, leaves with symptoms of a particular disease have been collected from different types of apple trees. • Images with various illumination conditions and heterogeneous backgrounds have been collected. • Images have been captured using cameras and other handheld devices having different optical characteristics.
Data Source Location	The data was collected from apple cultivation fields in different regions of Kashmir Valley.
Data Accessibility	Repository name: *Mendeley Data* Data identification number: doi:10.17632/9m2dcb5mmr.2 Direct URL to data: https://data.mendeley.com/datasets/9m2dcb5mmr/3 Instructions for accessing these data: *The images belonging to three classes are available in their individual directories*
Related Research Article	https://www.taylorfrancis.com/chapters/edit/10.1201/9781003405573-28/optimized-model-apple-leaf-disease-detection-performance-comparison-state-art-techniques-using-indigenous-dataset-arshad-ahmad-yatoo-amit-sharma

## 1 Value of the Data

• The dataset has been indigenously built to fit into the Indian context. Though there are some datasets available on Kaggle and other sources, they mainly belong to China and the Philippines and also lack authenticity.
• The dataset contains images in three classes viz. Healthy, Alternaria, Apple-Mosaic.
• Images have been collected from the natural fields and have been annotated by experts after proper data cleaning operation.
• This dataset is suitable for training deep-learning models for automated apple leaf disease detection.

## 2 Background

Several datasets are available in the public domain. As an example consider, PlantVillage [1] dataset containing more than 54000 images. The images belong to 14 crops with 38 class labels. The main disadvantage of this dataset is that it is unbalanced as the number of images is not uniform across classes within a particular crop. Moreover, the images in this dataset have been captured under controlled illumination conditions. Other datasets like [2,1] that are available in the public domain have the following disadvantages:

• Available datasets lack diversity as they have mostly been built by acquiring images under laboratory conditions and with a homogenous background.
• The images in the available data sets have been modified by removing the background.
• Apple leaves vary in size and shape depending on the apple variety. Existing datasets do not contain images across apple varieties.
• In some authentic datasets, the number of images per disease or class is much less. Datasets having small sizes in terms of the number of images do not adequately fit for the training of the CNN models.
• There are some authentic datasets belonging to China and the Philipines but as India is the 5^th^ largest producer of apples producing more than 2 metric tons of apples per year, a comprehensive and authentic dataset to deal with the local conditions is needed.

Due to the above reasons, the models that are trained on publicly available datasets do not perform well on unseen data in the actual field. This is called covariate shift, and this should not exist if a model has to perform well while addressing the issues of farmers.

The images in the proposed dataset have been collected from apple orchards using different devices and under varying environmental conditions. Image acquisition has been done on sunny days, rainy days, and cloudy days to incorporate diversity with respect to illumination conditions.

## 3 Data Description

**Fig. 1:**
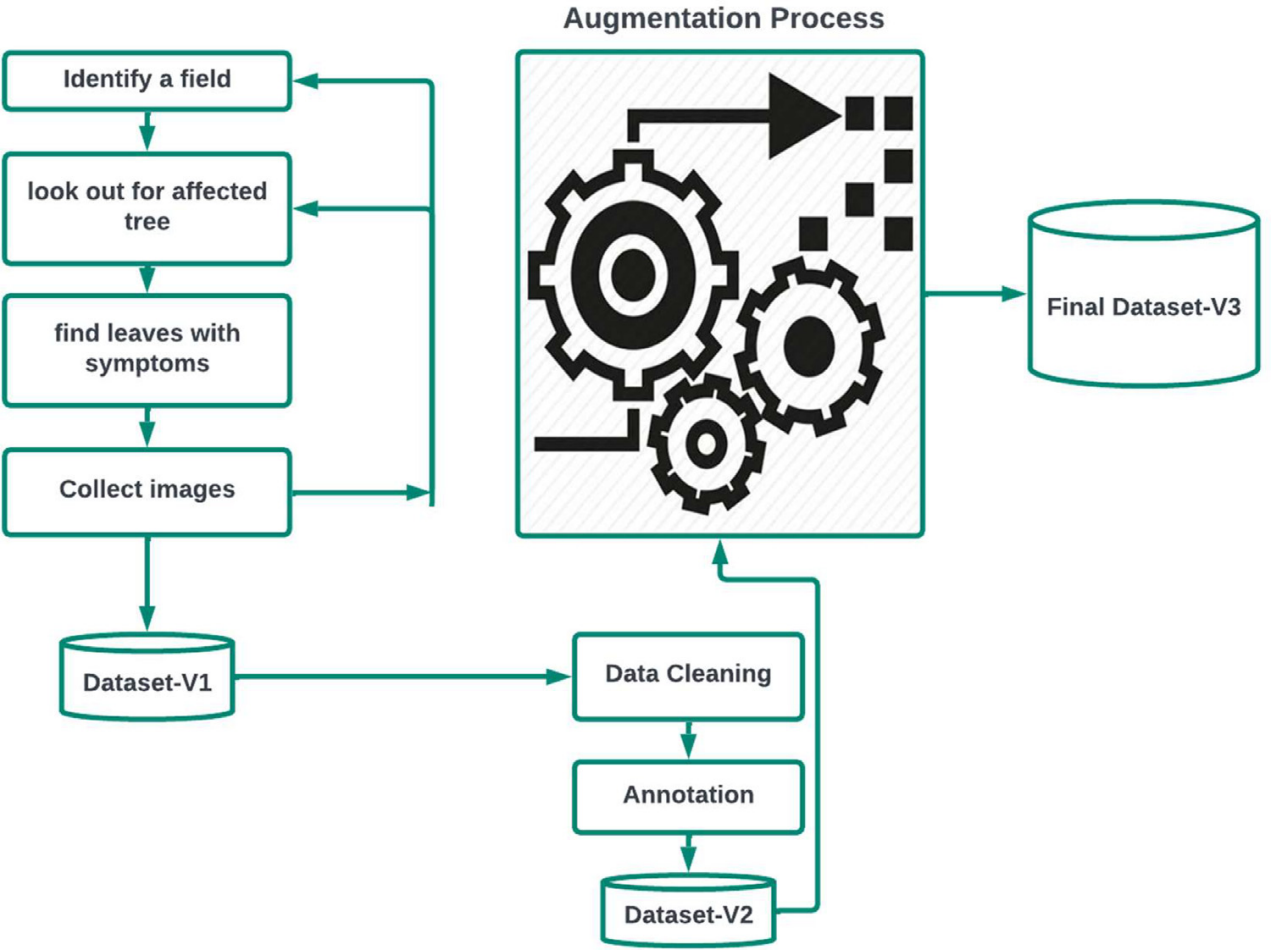
The procedure followed for building the dataset.

The following methodology, as shown in Fig. 1, was followed for building the dataset:

7569 Images in the dataset belong to three different classes, as per the details given in (Table 1). Different devices have been used to capture these images, and later, their size on disk and dimensions have been adjusted after annotation by the experts. Additionally, the image size (dimensions) have also been reduced to 256 × 256 pixels to make them suitable for most of the CNN models. While the parameters like size on disk and dimensions were reduced, the resolution was maintained at 96 dpi.

**Table 1:** Number of images in the dataset in different classes.

*S. No*	*Sample type*	*No. of Samples before augmentation*	*No. of Images* *After augmentation*	*Image type*	*Image Size*	*Dimensions*	*Horizontal/ Vertical Resolution*
1.	Healthy	740	2523	JPEG	12 KB	(256 × 256) pixels	96 dpi
2.	Alternaria	690	2523	JPEG	12 KB	(256 × 256) pixels	96 dpi
3.	Apple-Mosaic	725	2523	JPEG	12 KB	(256 × 256) pixels	96 dpi

The dataset has been split into Train_Set and Test_Set with a 70:30 ratio. Hence, the sub-folders in the dataset are:

*Train_Set****:*** Containing 1892 images of JPEG format in each category

*Test_Set****:*** Containing 631images of JPEG format in each category

**Fig. 2:**
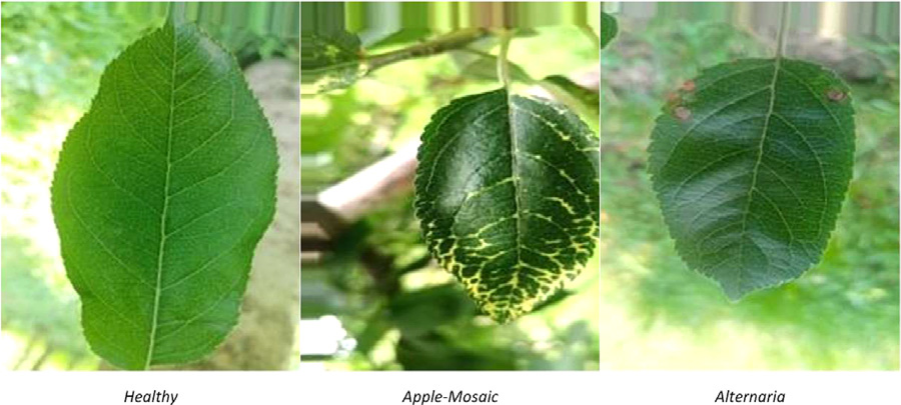
Sample from the dataset.

Fig. 2 shows sample images from the dataset

As foliar diseases develop symptoms on leaves, the images belonging to the Apple-Mosaic category have yellow lesions. These lesions diffuse throughout the leaves. As far as Alternaria is concerned, the symptoms are in the form of round-colored spots.

## 4 Experimental Design, Materials and Methods

The images were collected by the authors using different devices. Subsequently, an agriculturalist and disease expert carefully examined the region of interest (ROI) and classified the images. Some images were found to be having mechanical damage rather than lesions developed by foliar diseases. Such images were discarded. Finally, a phytopathology expert was consulted to check all the annotations to ensure accuracy. In such cases where decision-making was difficult, a majority vote including all experts’ suggestions was considered and annotations were corrected accordingly. Images collected were categorized into—Healthy, Apple-Mosaic and Alternaria. Image pre-processing techniques like resizing and contrast adjustment were applied through Python scripts on the data to improve the data quality.

### 4.1 Augmentation process

The dataset size has been increased through augmentation to prevent overfitting in deep learning models to be trained on the dataset. When a model performs well on training data, but its performance degrades on previously unseen data, this performance degradation is called overfitting [3]. Overfitted models tend to remember all the data, including inherent noise from the training data. Different augmentation techniques were used to create new samples to enlarge the dataset and infuse diversity. Augmentation techniques not only make a dataset bigger but also make it better [4] as these techniques improve data diversity, model robustness and translation invariance in addition to mitigating overfitting. Using the ImageDataGenerator class of the Keras library augmentation techniques like translation, rotation, shearing, image flipping, zooming, sharpening, and contrast variations were used to enlarge the dataset.

Augmentation techniques can be used on the fly or statically. The process of adding the enhanced data to the training dataset and utilizing it to train the model is known as static data augmentation. However, on the fly data augmentation is actually implemented when we randomly add data to each batch while the model is being trained. While on-the-fly augmentation is more resource-efficient in terms of computing and storage, data augmentation is more flexible when using the static approach [5]. For instance, we can personally review every generated sample while using static augmentation.

## Limitations

The dataset contains images belonging to three categories only. The dataset has a scope for further expansion to cover all identified diseases that impact the crop.

## Ethics Statement

The authors have read and followed the ethical requirements for publication in Data in Brief and confirm that the current work does not involve human subjects, animal experiments, or any data collected from social media platforms.

## CRediT Author Statement

All authors have equally contributed to the development of the dataset.

## Data Availability

Indigenous Dataset for Apple Leaf Disease Detection and Classification (Original data) (Mendeley Data)
